# Tablet-Based Automatic Assessment for Early Detection of Alzheimer's Disease Using Speech Responses to Daily Life Questions

**DOI:** 10.3389/fdgth.2021.653904

**Published:** 2021-03-17

**Authors:** Yasunori Yamada, Kaoru Shinkawa, Masatomo Kobayashi, Masafumi Nishimura, Miyuki Nemoto, Eriko Tsukada, Miho Ota, Kiyotaka Nemoto, Tetsuaki Arai

**Affiliations:** ^1^IBM Research, Tokyo, Japan; ^2^Department of Informatics, Graduate School of Integrated Science and Technology, Shizuoka University, Shizuoka, Japan; ^3^Department of Psychiatry, University of Tsukuba Hospital, Ibaraki, Japan; ^4^Department of Psychiatry, Faculty of Medicine, University of Tsukuba, Ibaraki, Japan

**Keywords:** language dysfunction, speech analysis and processing, mild cognitive impairment, Alzheimer's disease, health-monitoring, early screening

## Abstract

Health-monitoring technologies for automatically detecting the early signs of Alzheimer's disease (AD) have become increasingly important. Speech responses to neuropsychological tasks have been used for quantifying changes resulting from AD and differentiating AD and mild cognitive impairment (MCI) from cognitively normal (CN). However, whether and how other types of speech tasks with less burden on older adults could be used for detecting early signs of AD remains unexplored. In this study, we developed a tablet-based application and compared speech responses to daily life questions with those to neuropsychological tasks in terms of differentiating MCI from CN. We found that in daily life questions, around 80% of speech features showing significant differences between CN and MCI overlapped those showing significant differences in both our study and other studies using neuropsychological tasks, but the number of significantly different features as well as their effect sizes from life questions decreased compared with those from neuropsychological tasks. On the other hand, the results of classification models for detecting MCI by using the speech features showed that daily life questions could achieve high accuracy, i.e., 86.4%, comparable to neuropsychological tasks by using eight questions against all five neuropsychological tasks. Our results indicate that, while daily life questions may elicit weaker but statistically discernable differences in speech responses resulting from MCI than neuropsychological tasks, combining them could be useful for detecting MCI with comparable performance to using neuropsychological tasks, which could help develop health-monitoring technologies for early detection of AD in a less burdensome manner.

## 1. Introduction

As the world's elderly population increases, the number of people living with dementia is increasing rapidly, making dementia an increasingly serious health and social problem. According to World Alzheimer Report published in 2018, around 50 million people globally were living with dementia, corresponding to about 7.3% of the world's over-65-year-olds, and this number is projected to increase to 115 million by 2050 (1). Dementia affects not only individuals and their families but also the wider economy, with global costs estimated at about US$1 trillion annually, which is expected to increase to US$2 trillion by 2030 (2). Alzheimer's disease (AD) is the most common form of dementia and may account for an estimated 60–80 percent of cases (3, 4). Although no cure of AD is available, a growing body of evidence suggests that modifying risk factors could prevent or delay the onset of dementia including AD (5–7). From this perspective, there is an urgent need of early diagnosis at the early stages, e.g., mild cognitive impairment (MCI). However, diagnostic coverage worldwide remains so low that only 40–50% of people with dementia have been diagnosed even in high-income countries (8). In this context, health-monitoring technologies are expected to help in the early diagnosis of AD by detecting subtle changes in cognitive function from daily behaviors.

One of the clues for monitoring daily behaviors and detecting cognitive impairment due to AD might be speech data. While most typical hallmarks of both AD and MCI are deficits in memory and executive functions (9, 10), both retrospective analysis and prospective cohort studies have shown that language dysfunctions are also observed at the early stages of AD even from the presymptomatic period (11–14). Moreover, studies on pathologically proven AD patients showed that they exhibited syntactic simplification and impairment in lexical-semantic processing (15–18). A growing body of studies on probable AD patients has also shown that many aspects of speech and language, including linguistic characteristics such as grammatical and informational content as well as phonetic characteristics such as speech tempo and hesitation ratio, show deficits as AD progresses (13, 14, 19).

Previous computational studies attempted to measure these language and speech impairments in AD and MCI patients on the basis of such findings by using acoustic, prosodic, and linguistic features. For example, difficulties with word-finding and word-retrieving during verbal-fluency tasks have been measured by tallying pause frequency and fillers such as “umm” or “uh” (20–23). The reduction in speech expressiveness during picture-description tasks has also been quantified by measuring speech rate as well as the reduction in relevant information (18, 24–27). By using a combination of these features, previous studies have succeeded in differentiating AD and MCI patients from healthy controls (20, 21, 24, 25, 28–31). However, they mainly investigated speech data obtained while participants took part in neuropsychological tasks, typically conducted by clinicians. If we can detect language and speech impairments from other types of speech at sufficiently less burden such as responses to daily life questions such as regarding today's feeling and future travel plans, it would extend the scope of application to early detection of AD in various everyday situations.

There is growing interest in using speech data that can be collected from everyday situations for healthcare applications due to the expansion of mobile devices and voice-based interaction systems such as smartphones, tablets, and smart speakers. For example, mobile applications for collecting speech responses to neuropsychological tasks such as verbal fluency, counting backward, and picture description, have been developed and showed accurate classification rates for detecting patients with AD and MCI (32, 33). As other examples, vocal characteristics in speech data during typical tasks on smart speakers was suggested to be associated with cognitive scores of neuropsychological tests used for screening of dementia (34), while linguistic features extracted from conversational data of phone calls were indicated as significant indicators for differentiating AD patients from cognitively-normal (CN) older adults (35). These approaches focusing on speech data that can be collected from everyday situations would increase opportunities for assessment and help with the early detection of AD.

In this study, we developed a tablet-based application and investigated whether speech responses to daily life questions could be used to differentiate elderly patients with MCI from CN participants. For comparison, we also collected speech responses to neuropsychological tasks using the same tablet application and analyzed them. We first conducted statistical analysis to investigate speech features with significant differences between CN and MCI and compared them between responses to daily life questions and to neuropsychological tasks. By combining these speech features, we then constructed binary classification models for detecting MCI and compared their accuracies between the use of daily life questions and neuropsychological tasks. Through the analyses of both speech responses, we discuss how speech responses to daily life questions can be used for automatic tablet-based assessment for detecting MCI with less burden on older adults that is comparable in accuracy to using speech responses to neuropsychological tasks.

## 2. Methods and Materials

### 2.1. Participants

We recruited patients with MCI or dementia from University of Tsukuba Hospital, and other participants were the spouses of the patients and were recruited from local recruiting agencies and advertisement in the community in Ibaraki, Japan. All participants could speak Japanese and all examinations were conducted in Japanese. Participants were excluded if they had severe mental illness (major depression, bipolar disorder, schizophrenia), evidence of stroke affecting motor function, or poor command of the Japanese language. This study was conducted under the approval of the Ethics Committee, University of Tsukuba Hospital. We obtained informed written consent from all participants.

Irrespective of their diagnoses before attending the study, two psychiatrists of the authors (K. N. and T. A.) reviewed the patients' clinical notes and study assessments to verify their diagnoses. Cognitive performance of all participants was assessed using the following neuropsychological examinations conducted by neuropsychologists: the Mini-Mental State Examination (MMSE), immediate and delayed recall of the logical memory-story A of the Wechsler Memory Scale-Revised, the Frontal Assessment Battery, trail making test-part A and B, and clock drawing test. For clinical scales, all participants were administered the Geriatric Depression Scale and received a structural MRI scan using a three-dimensional magnetization prepared rapid gradient echo sequence. The Clinical Dementia Rating (CDR) for all patients was also used for diagnosis.

Regarding diagnosis of MCI and dementia, we applied standard research diagnostic criteria of the National Institute on Aging and Alzheimer's Association (NIA-AA) and AD Neuroimaging Initiative 2 (ADNI 2)^1^. Specifically, participants with MCI and dementia met NIA-AA criteria for MCI and all-cause dementia, respectively. The range of MMSE scores for the CN and MCI participants was 24–30 and for dementia patients was less than or equal to 26. The CDR global score for patients with MCI was 0.5 and for patients with dementia was more than 0.5. Delayed recall of the logical memory IIA subscale of the Wechsler Memory Scale-Revised was used with cutoff scores based on education: For participants with MCI and dementia patients, these scores were ≦ 11 and 8 for more than 16 years of education, ≦ 9 and 4 for 8–15 years of education, and ≦ 6 and 2 for 0–7 years of education, respectively.

The participants with CN and MCI were 76 Japanese seniors [35 females and 41 males; 61–87 years old; mean (SD) age: 71.9 (5.3) years]. The CN group consisted of 39 participants [22 females and 17 males; 61–80 years old; mean (SD) age: 70.1 (5.0) years] and the MCI group consisted of 37 participants [13 females and 24 males; 64–87 years old; mean (SD) age: 73.8 (5.0) years]. Table 1 shows clinical and demographic information.

**Table 1:** Demographics and clinical information of cognitively normal (CN) participants and those with mild cognitive impairment (MCI).

**Characteristics**	**CN (*n* = 39)**	**MCI (*n* = 37)**	**P value**
Age	70.1 (5.0)	73.8 (5.0)	<0.005
Female	22 (59.4%)	13 (35.0%)	0.063
Education [year]	13.2 (2.2)	13.7 (2.7)	0.306
MMSE [0–30]	27.9 (1.7)	27.2 (2.0)	0.089
LM			
I-A [0–25]	11.2 (3.4)	7.7 (3.4)	<0.001
II-A [0–25]	9.7 (2.8)	5.5 (3.3)	<0.001
FAB [0–18]	14.2 (2.2)	13.3 (3.0)	0.373
TMT			
Part A [s]	31.4 (8.6)	43.5 (15.9)	<0.001
Part B [s]	85.4 (41.9)	129.6 (68.1)	<0.001
CDT [0–7]	6.7 (0.9)	6.7 (0.6)	0.261
GDS [0–15]	2.9 (3.2)	3.6 (2.8)	0.207

*Data are presented as mean (standard deviations) or number (proportion %). P values were determined by using two-tailed Mann-Whitney U-tests for continuous data and χ^2^ test for categorical data. MMSE, Mini-Mental State Examination; LM, Logical Memory of the Wechsler Memory Scale-Revised; FAB, Frontal Assessment Battery; TMT, Trail Making Test; CDT, Clock Drawing Test; GDS, Geriatric Depression Scale*.

### 2.2. Speech Data Collection

**Figure 1:**
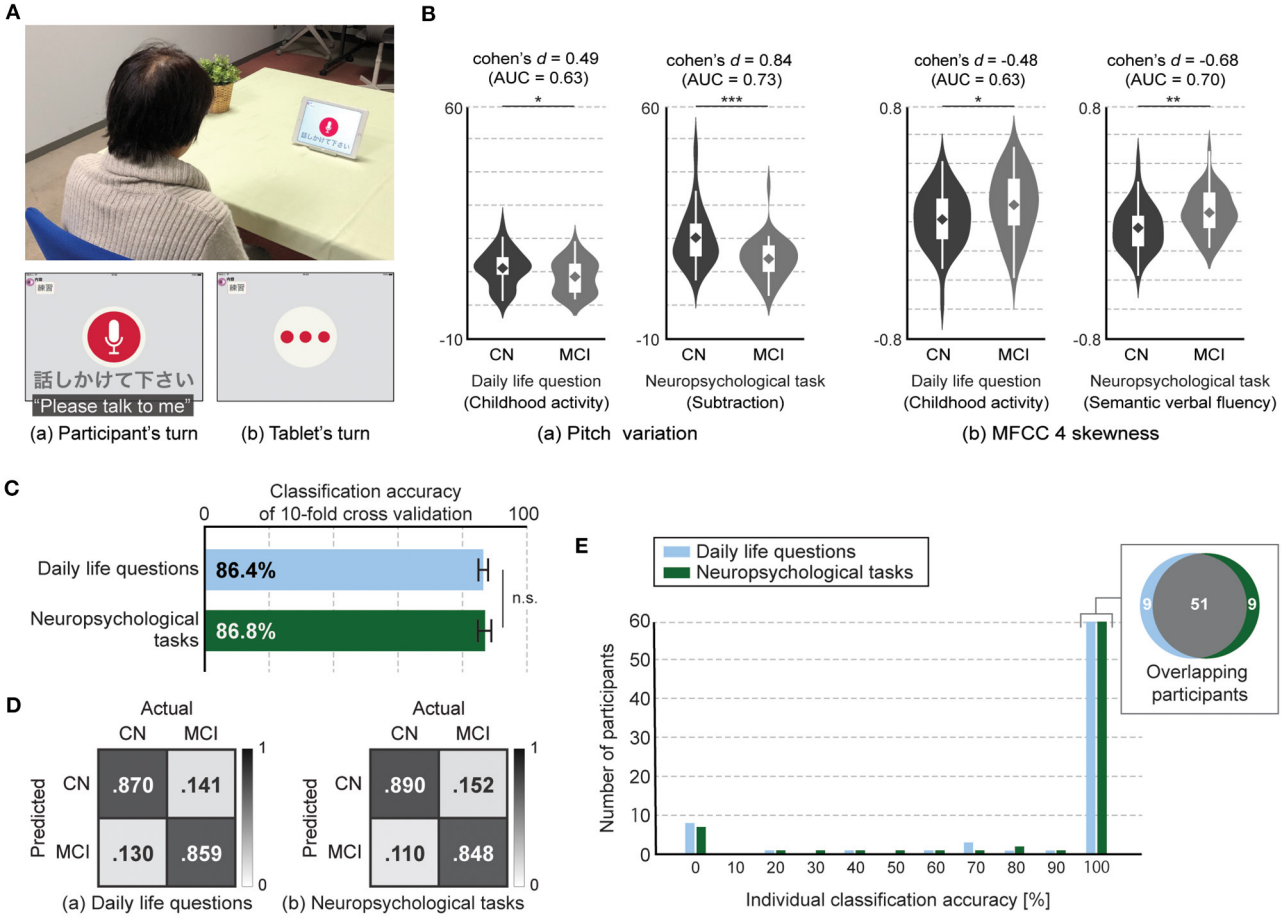
Tablet-based assessment using speech responses to daily life questions and neuropsychological tasks. **(A)** Experiment setup and application interface. **(B)** Speech features with statistical significances between CN and MCI participants in both types, i.e., daily life questions and neuropsychological tasks, (two-tailed Student's *t*-test with false discovery rate correction). Both speech features in responses to daily life questions had statistical significance but lower discriminative power between CN and MCI participants compared with those in responses to neuropsychological tasks. Significant differences are denoted with asterisks (*P < .05, **P < .01, ***P < .001). Violin plot is used to visualize distribution of data and probability density. On each side of violin is kernel-density estimation to show distribution shape of data. Wider portion of violin indicates higher density and narrow region represents relatively lower density. White box with whiskers in violin is boxplot. Box denotes 25th (Q1) and 75th (Q3) percentiles. Whiskers denote upper and lower adjacent values that are most extreme within Q3+1.5(Q3-Q1) and Q1-1.5(Q3-Q1), respectively. Line and diamond in box represent median and mean, respectively. **(C)** Accuracy of classification models for differentiating MCI from CN participants using features extracted from speech responses to daily life questions and neuropsychological tasks. Error bar represents standard deviation for 20 iterations of 10-fold cross validation. **(D)** Confusion matrices of classification models for differentiating MCI from CN participants using features extracted from speech responses to daily life questions and neuropsychological tasks. **(E)** Histogram of classification accuracy of individual participants. Gray area in Venn diagram represents number of overlapped participants who had 100% classification accuracy with both models using features extracted from speech responses to daily life questions and neuropsychological tasks.

Participants sat down in front of the tablet and answered questions presented by a voice-based application on the tablet. The tablet showed a screen indicating whether it was speaking or listening (Figure 1A). We used iPad Air and recorded voice responses by using an internal microphone of the iPad (core audio format, 44,100 Hz, stereo, 16-bit). Questions consisted of two categories: questions about daily life and tasks selected from neuropsychological tests.

The daily life questions consisted of eight questions designed to capture changes in memory and executive functions, in addition to language function, associated with MCI and dementia. Regarding memory function, three questions were related to episodic memory and asked participants to recall and explain old personal episodes about a fun childhood activity as well as recent episodes about what was eaten for dinner yesterday and the day before yesterday. In addition, one question was related to semantic memory regarding general knowledge and asked participants to explain a Japanese traditional event. Questions designed to capture executive function included two questions. One was related to planning and asked about response plans for an earthquake. The other was related to decision-making and asked participants to choose one option from among two regarding future travel destinations and to give three reasons for the choice. In addition to the above six questions, we used two questions frequently used in daily conversations, that is, how one feels today and one's sleep quality last night. For the actual sentences for the daily life questions, please see Supplementary Table 1.

The five neuropsychological tasks were, counting backward, subtraction, phonemic and semantic verbal fluency, and picture description with the Cookie Theft picture adapted from the Boston Diagnostic Aphasia Examination (36). These tasks were the most representative tasks used in previous studies on detecting AD from speech data. Note that these tasks were performed using the tablet and not included in the cognitive assessments for diagnosis conducted by neuropsychologists.

### 2.3. Data Analysis

Using the speech data, we investigated the feasibility of tablet-based assessments for early detection of AD by automatically extracting speech features and constructing binary classification models for differentiating participants with MCI from CN. We extracted 369 features consisting of 321 acoustic features and 48 prosodic features from responses to each task based on previous studies (see Table 2). For the binary classification model, we used support vector machine (SVM) models with feature-selection methods based on the area under the curve to avoid overfitting the models (45, 46). We evaluated the models with 20 iterations of 10-fold cross-validation.

**Table 2:** Number of questions showing statistical significance for each feature type between CN and MCI participants, and previous studies that reported significant differences in each feature type extracted from speech data during neuropsychological tasks.

**Feature type**	**Number of tasks** **showing statistical** **significance**		**Previous** **studies** **reported** **significant** **difference**	
	**Neuropsychological tasks**	**Daily life questions**		
MFCC	**5/5**	**8/8**	AD	(25, 37)
Pause	**3/5**	2/8	MCI	(38)
			AD	(39)
Pitch variation	**3/5**	1/8	AD	(23, 40)
Speech rate	**3/5**	1/8	AD	(23, 24, 40–42)
			MCI	(43)
Jitter	**3/5**	0/8	AD	(37, 40, 43)
			MCI	(43)
Shimmer	**3/5**	0/8	AD	(39, 43)
			MCI	(38, 43)
Phonation time	2/5	1/8	AD	(43, 44)
			MCI	(21)
Response time	1/5	2/8	AD	(21, 23)
Formant	2/5	**5/8**		Not found
Phoneme rate	2/5	1/8		Not found
Pitch	2/5	0/8		Not found

*Feature types that showed significant differences in more than half tasks are in bold*.

The acoustic features consisted of four feature types related to Mel-frequency cepstral coefficients (MFCCs), the first three formant frequencies (F1–F3), jitter (local, RAP, PPQ5, DDP), and shimmer (local, APQ3, APQ5, APQ11, DDA). We used the first 14 MFCCs, which represent the short-term power spectrum of the speech signal. The features of jitter and shimmer are measures of the cycle-to-cycle variations of fundamental frequency and amplitude (47). The prosodic features included seven feature types related to pitch and its variation, speech rate, phoneme rate, phonation time, pause, and response time. We calculated the statistics, i.e., mean, median, standard deviation, maximum, and minimum, of each feature type and used them as speech features. We also calculated the median absolute deviation of pitch and skewness and kurtosis of MFCCs. We used the Python-based audio processing libraries We used the Python-based audio processing libraries Librosa (48) and Signal_Analysis^2^ for extracting speech features.

We used the binary classification model for differentiating CN and MCI participants by using the speech features. Parameters that we searched were kernel functions including linear and radial basis function, the width of the radial basis function kernel, and the penalty parameter. We also carried out feature selection on the basis of area under the curve of receiver operating characteristic after removing highly correlated features. The number of features and the threshold for pair-wise correlation between features were also parameters. We conducted an exhaustive grid search by using average scores resulting from iterative 10-fold cross validation and determined the above parameters. We used the algorithm for the SVM implemented in MATLAB (MathWorks Inc., Natick, MA).

We evaluated model performance based on accuracy resulting from 20 iterations of 10-fold cross-validation. We also evaluated their classification accuracy for individual participant to measure how reliably each participant was classified across the nested models on the basis of previous studies (49, 50). We calculated the rate at which each participant was classified accurately through iterating 10-fold cross validation.

All statistical analyses were conducted in the MATLAB environment. A two-tailed Student's *t*-test with false discovery rate correction was used to assess differences in each variable between CN and MCI participants. The *P* values below 0.05 were considered significantly different. Effect size was calculated using Cohen's *d*. We categorized the magnitude of Cohen's *d* in accordance with a previous study: small (0.2 ≦ *d* < 0.5), medium (0.5 ≦ *d* < 0.8), and large (*d* ≧ 0.8) (51).

## 3. Results

We obtained an average of 319.7 s (SD: 108.5) of speech responses to the eight daily life questions and 261.4 s (SD: 48.5) of speech responses to the five neuropsychological tasks using the tablet. The average duration of responses to each question varied, and the ranges were 4.2–75.4 s for the daily life questions and 32.5–73.1 s for the neuropsychological tasks (Supplementary Table 2). There was no significant difference in response durations to each question between CN and MCI participants (*p* > 0.05, Two-tailed Mann–Whitney *U*-test, Supplementary Table 2).

We first investigated whether and how the speech features differed between the CN and MCI participants. We found that among the 369 speech features, the average number of significant different features for each question was 8.4 (SD: 4.6; range: 3–16) for the eight daily life questions and 29.0 (SD: 10.2; range: 15–40) for the five neuropsychological tasks, respectively (Figure 1B). The top six questions with the highest number of significant features included all five neuropsychological tasks and one daily life question regarding future travel plans (Supplementary Table 3). We also found a similar trend in the effect size of each feature (Supplementary Table 3). The average number of features with more than medium effect size was 6.0 (SD: 3.1; range: 2–11) for the eight daily life questions and 20.0 (SD: 9.5; range: 9–33) for the five neuropsychological tasks, and the top six questions with the highest number included all five neuropsychological tasks and one daily life question regarding future travel plans. In addition, the features with large effect sizes were observed only in three neuropsychological tasks: subtraction, counting backward, and picture description. Therefore, we found that the number of significantly different features between CN and MCI participants as well as their effect sizes decreased in responses to daily life questions compared with those to neuropsychological tasks.

We next investigated what types of speech features showed significant difference between CN and MCI participants. We first compared significantly different feature types in speech responses to the neuropsychological tasks with those in previous studies on speech data during neuropsychological tasks. Previous studies reported the following eight types of speech features as statistically significant measures for MCI and/or AD: MFCCs, pause, pitch variation, speech rate, shimmer, jitter, phonation time, and response time (see Table 2). Among these eight feature types, we found that six showed significant differences in more than half the neuropsychological tasks in our study: MFCCs in all five tasks, pause, pitch variation, speech rate, shimmer and jitter in three out of the five tasks, and phonation time and response time in two and one out of the five tasks, respectively (Table 2). On the other hand, speech responses to daily life questions had 52 out of the 369 speech features with significant differences between CN and MCI participants, and 41, i.e., 78.8%, corresponded to speech-feature types showing significant differences in both this study and other studies using responses to neuropsychological tasks. Specifically, these 41 features consisted of 30 related to MFCCs, 3 related to pause, 1 related to pitch variation, 2 related to speech rate, 1 related to response time, and 4 related to phonation time. Features related to MFCCs were significantly different feature types in all responses to both daily life questions and neuropsychological tasks investigated in this study. In addition to MFCCs, formant features showed significant differences in more than half the daily life questions, but this feature type was not included in the significantly different feature types in speech responses to the neuropsychological tasks based on previous studies. In contrast, speech features related to shimmer and jitter extracted from responses to neuropsychological tasks showed significant measures for MCI and/or AD in both our study and previous studies, but they showed no significant differences in responses to all eight daily life questions.

We next constructed binary classification models for differentiating the MCI from CN participants with feature-selection methods and evaluated them with 20 iterations of 10-fold cross-validation. The model using speech responses to daily life questions achieved 86.4 ± 1.5% accuracy (85.9% sensitivity, 87.0% specificity, 86.1% F-measure), while the model using responses to neuropsychological tasks achieved 86.8 ± 2.1% accuracy (84.8% sensitivity, 89.1% specificity, 86.8% F-measure). We did not find significant difference between these two accuracies (*p* = 0.5, Student's *t*-test; Figures 1C,D). We also found that classification accuracy of the model using responses to seven out of the eight daily questions was up to 84.7 ± 1.2% accuracy, which was significantly lower than that using responses to neuropsychological tasks (*p* < 0.0005, Student's *t*-test).

Finally, we investigated classification accuracy for individual participants and found that 78.9% of all participants (60 out of 76) had 100% individual accuracy in both speech data related to daily life questions and neuropsychological tasks. Fifty-one participants overlapped, that is, 9 participants each showed 100% individual accuracy in only 1 of the 2 task types, i.e., daily questions and neuropsychological tasks, (Figure 1E). In addition, four out of both nine participants each showed less than 50% individual accuracy for the model using the speech responses to other types.

## 4. Discussion

In response to the increasing demand for early detection of AD, we investigated the possibility of tablet-based automatic assessment using speech data. Although previous studies have succeeded in differentiating MCI and AD patients from cognitive normal older adults by using speech responses to neuropsychological tasks, whether and how other types of speech responses to such tasks with sufficiently less burden could be used for detecting early signs of AD has not been sufficiently investigated. We developed a tablet-based application and collected speech responses to daily life questions, such as regarding today's feeling and future travel plans, from 76 Japanese seniors including 37 patients with MCI. We then investigated whether these responses could be used to differentiate MCI and CN participants through a comparison of speech responses to neuropsychological tasks conducted with the same tablet.

The statistical analysis showed that daily life questions could elicit discernable differences between CN and MCI participants in speech responses, although the number of features with significant differences and their discriminative power might decrease compared with those from neuropsychological tasks. This may be explained using neuropsychological tasks designed to measure subtle changes by imposing a heavy load on target cognitive domains; thus, speech features extracted from their responses could show a more discernable difference in accordance with the degree of cognitive impairment compared with other types of speech responses (52–54). Daily life questions, such as those regarding last night's dinner, response plan for a disaster, and future travel plans, are also associated with several cognitive functions including memory, although they might impose lighter cognitive loads than neuropsychological tasks (53, 55). Previous studies on daily conversations among AD patients and CN individuals reported several language and speech changes considered caused by cognitive impairments (35, 55, 56). Our results indicate that daily life questions can induce subtle but statistically detectable changes in speech features according to cognitive impairment due to AD even at early stages such as MCI. Our results also indicate that around 80% of speech features with significant differences correspond to speech-feature types that showed significant differences in responses of CN participants and MCI/AD patients to neuropsychological tasks. This also supports our hypothesis that daily life questions can elicit responses' changes associated with cognitive impairments resulting from AD.

We also reported similarities and differences in speech-feature types with significant differences between CN and MCI participants between daily life questions and neuropsychological tasks. We found that MFCCs were the most robust and showed significant differences in all responses to both daily life questions and neuropsychological tasks. Many studies suggested the MFCC is a good representation for detecting various types of diseases including depression, Parkinson's disease, and AD (25, 57, 58). One of our contributions lies in providing the first empirical evidence that MFCC-based features can be used for detecting early stages of AD from speech responses to not only neuropsychological tasks but also daily life questions. We found that feature types related to pause, pitch variation, speech rate, shimmer, and jitter tended to show statistical differences more in responses to neuropsychological tasks, while feature types related to formant frequencies tended to show more in responses to daily life questions. Neuropsychological tasks, such as verbal fluency, counting backwards, and picture description, typically encourage responses with many words and/or as fast as possible. This might be one of the reasons that speech features related to pause, speech rate, and phonation time tended to show more significant differences in responses to neuropsychological tasks than in those to daily life questions.

Results of evaluating binary classification models for detecting MCI showed that daily life questions could achieve high accuracy, comparable to neuropsychological tasks by using more responses to daily life questions (eight) than responses to neuropsychological tasks (five). The need for more questions about daily life might be explained by the smaller number of speech features with significant differences and smaller effect sizes than those in neuropsychological tasks. This might also suggest the trade-off between the degree of cognitive workload of each question and the number of questions needed to achieve the target classification performance, although this relationship may be more complex; for example, adding questions with similar profiles of cognitive workload might have little effect on classification performance. Moreover, the average response duration to all eight daily life questions was about 320 s in our study, which means 61 s longer than the response duration to the five neuropsychological tasks. Thus, the impact of the additional questions did not seem to be large, and the response duration was still not long compared with typical neuropsychological tests in spite of high detection accuracy. From this perspective, daily life questions might be easier to use regularly with less burden on older adults than neuropsychological tasks and may be a promising approach for timely and early detection of AD by frequent tablet-based automatic assessments. In addition, this approach may be useful for other neurodegenerative diseases and mental illnesses. For example, cognitive impairment is commonly observed in non-demented Parkinson's disease patients, and its early detection and intervention have been needed to prevent progressive cognitive decline and dementia (59–61). In bipolar disorders, cognitive impairment has been suggested as a predictor related to a worse clinical course and poorer functional outcome (62–64). Therefore, our approach of frequent automatic assessments focusing on daily life conversations may provide a useful tool for the early detection of cognitive impairments in patients with other diseases.

Results of classification accuracy for individual participant showed that both classification models using daily life questions and neuropsychological tasks could achieve high reliability for classification of each individual participant: 78.9% of the participants were classified correctly 100% of the time (100% individual accuracy). We also found that 11% of participants (8 out of the 76 participants) showed 100% individual accuracy on one of the task types (daily life questions or neuropsychological tasks) but less than chance level on the other. This suggests that combining both types could improve the accuracy of a binary classification model regarding each individual.

There were several limitations in this study. First, we collected speech data in a lab setting. The controlled setting might influence how people respond to questions. Therefore, we need to investigate speech responses collected in various living situations, such as at home, by using our tablet-based application. Second, the number of questions was small and limited. Although our study provided the first empirical evidence of the usefulness of daily life questions for detecting early stages of AD, what types of daily life questions that could elicit discernable changes related to AD remains uninvestigated. To achieve this, data collection at home would be good way to collect many speech responses by having participants use applications on a daily basis. Third, we did not directly evaluate the usability of the tablet-based application with the daily life questions. In a future *in-situ* study, we will evaluate how many and what kinds of daily life questions are acceptable for older adults to answer on a daily basis. Fourth, the number of participants was limited, although we decided the minimum sample size on the basis of previous studies on speech analysis for MCI and AD (54, 65). The power of the *post-hoc* power analysis for our results exceeded 0.90, but given the wide clinical profile of MCI and AD, a larger study on more participants may be needed to confirm our results. Fifth, the results were obtained by analyzing speech data in Japanese. We thus need to investigate speech data in other languages to confirm our results regarding the usefulness of speech responses to daily life questions for early detection of AD. In this study, we used only acoustic and prosodic features without linguistic features. Our analysis results of speech responses to neuropsychological tasks in Japanese were consistent with those in previous studies on speech responses in English. Therefore, we believe that our results will be confirmed in other languages.

In summary, we investigated the possibility of using speech responses to daily life questions for tablet-based automatic assessments for detecting MCI through by comparing them with those to neuropsychological tasks. We argued that daily life questions can elicit weaker but statistically discernable differences in speech responses associated with MCI than neuropsychological tasks. In addition, a classification model using the daily life questions could detect MCI with high accuracy, statistically comparable to that using neuropsychological tasks. We believe that our results can help promote future efforts toward early detection of AD by using speech responses to various types of less burdensome questions.

## Data Availability Statement

The datasets presented in this article are not readily available, but derived and supporting data may be available from the corresponding authors on reasonable request and with permission from the Ethics Committee, University of Tsukuba Hospital. Requests to access the datasets should be directed to Yasunori Yamada, ysnr@jp.ibm.com; Kaoru Shinkawa, kaoruma@jp.ibm.com.

## Ethics Statement

The studies involving human participants were reviewed and approved by Ethics Committee of University of Tsukuba Hospital. The patients/participants provided their written informed consent to participate in this study.

## Author Contributions

YY, KS, MK, and MNi contributed to conception and design of the study. YY, KS, and MNe conducted the experiments. ET, MO, KN, and TA contributed to the recruitment of patients and diagnosis for participants. YY and KS performed the analysis and wrote the manuscript. All authors have approved the final version of this manuscript.

## Conflict of Interest

YY, KS, and MK are employed by the IBM Corporation. The remaining authors declare that the research was conducted in the absence of any commercial or financial relationships that could be construed as a potential conflict of interest.

## Footnotes

^1^ https://adni.loni.usc.edu/wp-content/uploads/2008/07/adni2-procedures-manual.pdf

^2^ https://brookemosby.github.io/Signal_Analysis/
