# Supplementary material for: Tablet-Based Automatic Assessment for Early Detection of Alzheimer's Disease Using Speech Responses to Daily Life Questions
Source: Front Digit Health. 2021 Mar 17;3:653904. doi: 10.3389/fdgth.2021.653904 (PMC8521899; doi:10.3389/fdgth.2021.653904)
Supplement: Supplementary file 1 [file Data_Sheet_1.pdf]

Supplementary table 1. Actual sentences of daily life questions (translated from Japanese).

Daily life questions	Actual sentences
A fun childhood activity	"Please explain one of the fun activities you engaged in when you were a child."
Dinner menu for yesterday	"What did you eat for dinner yesterday ?"
Dinner menu for the day before yesterday	"What did you eat for dinner the day before yesterday?"
Japanese traditional event	"If you could explain the Shichi-Go-San to someone who doesn't know about the event, how would you explain it?"
Response plan for an earthquake	"If an earthquake strikes while you are at home, how would you act and in what order?"
Future travel destination	"If you could go on a trip this summer, where would you like to go, the mountains or the sea? Please choose one and give three reasons why you chose it."
Today's feeling	"How are you doing today ?"
Sleep quality last night	"Did you sleep well last night?"

Supplementary table 2. Duration of speech data of each neuropsychological task and daily life question. Data are presented as mean (standard deviations).

	Neuropsychological task/Daily life question	Total (n=76) [sec]		Cognitively normal (CN) (n = 39) [sec]		Mild cognitive impairment (MCI) (n = 37) [sec]		*P* Value*
**Neuropsychological task**	Counting backward (Count down from 305 to 290)	32.5	(9.1)	31.0	(6.3)	34.1	(11.2)	0.240
	Subtraction (Count down from 925 by threes)	43.3	(22.0)	39.0	(19.0)	47.9	(24.2)	0.064
	Semantic verbal fluency task (Produce as many animal names in 60 seconds)	59.5	(6.5)	60.0	(4.5)	59.0	(8.1)	0.655
	Phonemic verbal fluency task (Produce as many noun words beginning with Japanese mora “ta” in 60 seconds)	53.0	(10.4)	51.3	(10.5)	54.8	(10.1)	0.180
	Picture description (Describe characters and events in the picture†)	73.1	(31.5)	66.1	(25.3)	80.4	(35.8)	0.074
**Daily life question**	Today’s feeling	4.4	(5.5)	5.0	(6.7)	3.7	(3.9)	0.271
	Sleep quality last night	4.2	(4.2)	4.3	(3.8)	4.0	(4.7)	0.536
	Dinner menu for yesterday	21.8	(19.6)	23.3	(19.0)	20.2	(20.2)	0.230
	Dinner menu for the day before yesterday	21.0	(19.9)	24.5	(21.2)	17.2	(18.0)	0.058
	A fun childhood activity	75.4	(39.8)	74.9	(40.1)	75.8	(40.0)	0.755
	Japanese traditional event	68.1	(28.8)	69.9	(33.0)	66.3	(24.0)	0.967
	Future travel destination	57.6	(26.6)	59.1	(26.1)	56.0	(27.3)	0.561
	Response plan for an earthquake	67.3	(33.2)	70.9	(32.1)	63.4	(34.3)	0.161

* Two-tailed Mann-Whitney U Test

† The Cookie-Theft Picture Task, adapted from the Boston Diagnostic Aphasia Examination (Goodglass H, 1983)

Supplementary table 3. Proportion of significant features and features with medium/large effect size for each neuropsychological task and daily life question. Significant feature was counted if *P* value of two-sided t-test with false discovery rate multiple testing correction was less than 0.05. Feature with medium effect size was counted if Cohen's *d* was greater than 0.5. Feature with large effect size was counted if Cohen's *d* was greater than 0.8.

	Neuropsychological task/Daily life question	Proportion of significant features		Proportion of features with medium effect size		Proportion of features with large effect size	
Neuropsychological task	Subtraction (Count down from 925 by threes)	0.8%	(40/369)	8.9%	(33/369)	0.5 %	(2/369)
	Picture description (Describe characters and events in the picture†)	10.0%	(37/369)	7.0%	(26/369)	0.3%	(1/369)
	Semantic verbal fluency task (Produce as many animal names in 60 seconds)	8.1%	(30/369)	4.6%	(17/369)	0%	(0/369)
	Counting backward (Count down from 305 to 290)	6.2%	(23/369)	4.0%	(15/369)	0.5%	(1/369)
	Phonemic verbal fluency task (Produce as many noun words beginning with Japanese mora "ta" in 60 seconds)	4.1%	(15/369)	2.4%	(9/369)	0%	(0/369)
Daily life question	Future travel destination	4.3%	(16/369)	3.0%	(11/369)	0%	(0/369)
	A fun childhood activity	3.3%	(12/369)	1.9%	(7/369)	0%	(0/369)
	Japanese traditional event	3.3%	(12/369)	2.4%	(9/369)	0%	(0/369)
	Response plan for an earthquake	2.4%	(9/369)	1.9%	(7/369)	0%	(0/369)
	Today's feeling	1.6%	(6/369)	1.4%	(5/369)	0%	(0/369)
	Sleep quality last night	1.4%	(5/369)	0.8%	(3/369)	0%	(0/369)
	Dinner menu for yesterday	1.1%	(4/369)	1.0%	(4/369)	0%	(0/369)
	Dinner menu for the day before yesterday	0.8%	(3/369)	0.5%	(2/369)	0%	(0/369)

† The Cookie-Theft Picture Task, adapted from the Boston Diagnostic Aphasia Examination (Goodglass H, 1983)
